# Notes on current Mbyá-Guarani medicinal plant exchanges in southern Brazil

**DOI:** 10.1186/s13002-021-00465-w

**Published:** 2021-06-02

**Authors:** Julian Henrique Carlotto de Andrade, José Rodrigues, André Benites, Cornélio Benites, Arlindo Acosta, Marcelina Benites, Cocelina Benites, Ilda Gomes, Jaime Valdir da Silva, Eunice Antunes, Elisete Antunes, José Martins, Daniel Martins Timóteo, Santiago Franco, José Cirilo Pires Morinico, Fernanda Ribeiro da Silva, Natalia Hanazaki

**Affiliations:** 1 Programa de Pós-Graduação em Biologia de Fungos, Algas e Plantas, Centro de Ciências Biológicas, Universidade Federal de Santa Catarina, Florianópolis, SC 88010-970 Brazil; 2 Laboratório de Ecologia Humana e Etnobotânica (ECOHE), Centro de Ciências Biológicas, Universidade Federal e Santa Catarina, Florianópolis, SC 88010-970 Brazil; 3 Tekoá Nhuu Porã, Terra Indígena Campo Molhado, Florianópolis, SC Brazil; 4 Tekoá Ka’aguy Porã, Terra Indígena Retomada, Florianópolis, SC Brazil; 5 Tekoá Jatai’ty, Terra Indígena Cantagalo, Florianópolis, SC Brazil; 6 Tekoá Itaty, Tekoá Yakã Porã, Terra Indígena Morro dos Cavalos, Florianópolis, SC Brazil; 7 Tekoá Yyn Moroty Vherá, Terra Indígena Mbiguacu, Florianópolis, SC Brazil; 8 Tekoá Ywy Poty, Terra Indígena Flor da Terra, Florianópolis, SC Brazil; 9 Tekoá Anhetengua, Terra Indígena Lomba do Pinheiro, Florianópolis, SC Brazil

**Keywords:** Ethnobotany, Mbyá-Guarani, Exchange networks, Traditional medicine

## Abstract

**Background:**

Experts in the Atlantic Forest, the Guarani people have the habit of transporting and exchanging plants due to their mobility throughout the territory. Historically, this habit contributed to the species composition and diversification among different phytophysiognomies that comprise the Atlantic Forest. Medicine and spirituality are traits that stand out within the Guarani culture, which is based on a holistic understanding of physical and spiritual well-being for the person’s health. To achieve this balance, they use a range of native and adapted plant species. Our goal is to understand some of the Guarani contributions to the cultural landscape in the Atlantic Forest.

**Methods:**

We conducted semi-structured interviews with key persons asking about the importance of forest environments for Guarani health and about visits to other Guarani villages and plants exchanged. Data analysis was qualitative, and through a bipartite network of exchanged plants to show current plant exchanges between villages.

**Results:**

We visited seven Guarani Indigenous Territories in south Brazil, and with the participation of 12 respondents, we registered 27 species that were exchanged through different phytophysiognomies in the Atlantic Forest. These results show an intense movement of plants currently occurring between villages and the importance of these movements for both individual health and the integrity of the environments in which the Guarani villages are inserted.

**Conclusions:**

We observed a search for the maintenance of traditional species in the Guarani medical system, and we highlight the fundamental role of Guarani management in the conservation of the southern Atlantic Forest in indigenous territories.

## Background

Since immemorial times, the use of medicinal plants is the best known and most practiced way to treat and relieve diseases among local communities, such as indigenous peoples [1, 2]. In Brazil, there are among 305 indigenous peoples, and the Guarani are one of the most populous of them, with approximately 85,000 people [3]. The Guarani are currently divided into three main groups: Kaiowá, Nhandevá/Chiripá, and Mbyá, the latter corresponding to the majority of the Guarani population living near the Brazilian coast [4, 5].

Historically, the Guarani occupy the lowland region of South America, mainly in the Paraguay, Paraná, and Uruguay River basins, and the southern Atlantic coast of Brazil, ranging from the states of São Paulo to Rio Grande do Sul, where they are currently distributed. The Guarani also inhabit the Brazilian states of Mato Grosso do Sul, Espírito Santo, Rio de Janeiro, Tocantins, and Pará. With the exception of these last two states, the Guarani territories are within the Atlantic Forest biome, which is a fundamental element for the Guarani way of being, the *nhandereko*. Along many centuries of occupation of this biome, the Guarani people established deep relations with the forest. The Guarani presence in the region is estimated, from archeological records, to be at least ca. 2000 years, depending on location [6–10]. Unfortunately, the demarcation of their territories is still one of the major challenges faced by the Guarani, despite being the native inhabitants of the region.

Ethnographic research about the Guarani people includes studies on their sociocultural identity, both historical and contemporaneous migratory processes, and botanical, agroecological, and ecological knowledge that report their vast knowledge of the native flora [11–15]. Being descendants of the Tupi cultural matrix, the Guarani continued the agroforestry systems developed in the Amazon, in their migration and expansion movements and in a process of ecological adaptation, seeking fertile areas in the subtropical forests that best fit their lifestyle ([7, 10, 16]; see also [13, 17–19]). Thus, linked to their plant knowledge, another culturally striking feature that accompanied the Guarani throughout their history is their mobility.

The Guarani are dynamic people. Visits and migrations between villages are part of their way of life. Visits favor exchanges and are related to the strengthening of family ties, bringing health and happiness, and by economic-environmental or cosmological aspects [20–22]. Many indigenous peoples have a systemic and holistic perception of the world [23], with no boundaries between humans (society) and nature (ecosystem) [24]. In Guarani cosmology, moving through the traditional territory (*Yvy Rupá*) is part of their well-being, or *teko porã*, maintaining joy and health [25]. The principle of reciprocity, or *mborayu*, is fundamental for the Mbyá-Guarani [20]: guided by kinship, economic activities, and other aspects of social life such as residence, religious life, alliances, and leaderships, this principle assumes varied features that are related to their own mobility [14].

Thus, Guarani mobility is also related to the transportation and exchange of plants [1, 5, 16, 26–28]. This mobility contributes to the availability and diversity of food plants and of the Guarani pharmacopoeia [5, 13, 27] and can contribute to the formation of cultural landscapes [29–31] within the Atlantic Forest. The Atlantic Forest inhabited by the Guarani includes different phytophysiognomies, comprising areas of dense ombrophylous forest, mixed ombrophylous forest, seasonal semideciduous forest, and deciduous forest, as well as transition areas between these phytophysiognomies and areas in different successional stages [10, 21, 27, 32]. The circulation and transport of propagules between different villages may result in exchanges of plants among these different phytophysiognomies.

According to Votre et al. [32], the success of Guarani occupations in forest environments is due to their botanical and ecological knowledge. A database of useful species of the flora for the Guarani in Santa Catarina (southern Brazil), Argentina, and Paraguay, organized by Pereira, Noelli, Campos, Santos, Zocche [28] and complemented by Votre et al. [32], registered the traditional use of 956 species belonging to 131 botanical families. Noelli [18] surveyed plants for therapeutic purposes among Guarani communities of Paraguay, Mato Grosso do Sul, Argentina, and Rio Grande do Sul and listed 151 species from an inventory of more than 800 plants. Additionally, Crovetto [33] cited the use of 438 plants among Mbyá in northwestern Argentina, most for medicinal purposes.

The circulation and transport of plants and seeds and the associated transmission of knowledge can be studied through social network analysis [34–37]. These studies highlight variations in traditional ecological knowledge related to characteristics such as age, gender, kinship, education, place of residence, social position, and level of integration in the economic market. For example, in two Tsimane villages in the Bolivian Amazon, Díaz-Reviriego et al. [34] found high rates of diversity in exchange networks of home-grown crops, influenced by women and kinship ties. Similarly, Lope-Alzina [37] observed that among members of a Yucatec-Maya community in Mexico, home gardens are the main source of exchanged plants. Despite strong market share, gifts remain the predominant form of exchange, with most gifts coming from home gardens and with most exchanges taking place among women in kinship networks [37]. Routes of knowledge transmission about medicinal plants among the Yucatec-Maya in Mexico showed that the individual knowledge of medicinal plants is positively associated with the position in the network of exchanges of knowledge about herbal medicines [36]. In Africa, Europe, Latin America, and Oceania, Coomes et al. [38] showed that farmers with leading social positions and strong ethnobotanical knowledge were expected to be the main seed suppliers in seed networks.

The aim of this study was to investigate current exchanges of plants between Guarani villages, contributing to the formation of cultural landscapes [13, 29, 31], which may have occurred since pre-Columbian times [30]. The specific objectives were (1) to describe the exchange networks of plant propagules of medicinal and healing importance between Guarani villages in southern Brazil, and (2) to discuss the importance of plants and environments for eco-cultural Guarani health. We focus on seeking plants used and interchanged for medicinal or healing purposes (here, we are using the term healing to encompass magic and ritualistic plants, since medicine and spirituality are intertwined in Guarani worldview), including plants used in the treatment of illnesses of the spirit or in magical contexts, such as spells and magic (*ka'avo*), according to the traditional healing practices, also supported by Guarani bibliography [27].

## Material and methods

### Study area

Seven Mbyá Guarani Indigenous Lands in the Atlantic Forest of southern Brazil were included in this research. They were chosen by convenience due to pre-existing contacts of the first author and the indication of propagule exchanges among them. The collaborators from one village were asked to indicate other villages with whom they exchange plants, and thus we started to build the contacts and to discuss prior informed consent with the leaders of these other villages to include them in the sampling. The Atlantic Forest is a hotspot for biodiversity conservation [39], and the Guarani villages included in this study are in different phytophysiognomies (sensu of Instituto Brasileiro de Geografia e Estatística [40]): Dense Ombrophylous Forest, Seasonal Semideciduous Forest, Dense Ombrophylous Forest, and Mixed Ombrophylous Forest. The Indigenous Lands are: (1) *Nhuu Porã*, (2) *Ka'aguy Porã*, (3) *Yvy Poty*, (4) *Anhetenguá*, (5) *Jatai’ty*, (6) *Itaty* and *Yakã Porã*, and (7) *Yyn Moroty Vherá* (see details on Table 1).

**Table 1:** General characteristics of the seven Guarani villages included in this study

Village	Indigenous land	Number of families	Size	Location	Legal recognition	Characteristics
*Nhuu Porã*	Campo Molhado	5–6	2268 ha	Riozinho, Maquiné, and Caraá	2001	DOM/MOF in medium to advanced regeneration
*Ka'aguy Porã*	Retomada	12	367 ha	Maquiné	2017 (occupation)	DOF
*Yvy Poty*	Flor da Terra	12	100 ha	Barra do Ribeiro	2014 (acquired)	SSF
*Anhetenguá*	Lomba do Pinheiro	16	25 ha	Porto Alegre	2012	SSF fragments but mostly *Pinus* sp.
*Jatai’ty*	Cantagalo	52	283.67 ha	Viamão	2007	SSF and areas with *Pinus* sp
*Itaty* and *Yakã Porã*	Morro dos Cavalos	37	1998 ha	Palhoça	1008	DOF
*Yyn Moroty Vherá*	Mbiguaçu	44	59 ha	Biguaçu	2003	DOF

*DOM* dense ombrophylous forest, *SSF* seasonal semideciduous forest, *MOF* mixed ombrophylous forest

### Data collection

Data were collected between October 2017 and November 2018. Whenever possible, the visits to each village were accompanied by a person from a former village where we did interviews. Considering the participation of Guarani interviewees as active collaborators in this study, they were invited to be co-authors. Two chiefs who accompanied the first author in the interviews in their own village and in other Indigenous Territories were also invited to be co-authors. The maximum length of stay in one village was 1 week, but in two villages, the visit was only 1 day; however, the arrangements to visit each village took longer due to the previous contacts with the leaders, the discussion and acceptance of the prior informed consent, and the arrangements of the visit. The length of stay in each village depended on the availability of the people to be interviewed. Besides the seven Indigenous Territories visited in this study, we tried to visit another five, with no success, due to the unavailability of collaborators to accompany the first author or due to difficulties of scheduling appropriate moments with the leaders to discuss prior informed consent. All interviews were preceded by obtaining prior consent from village leaders through agreement with a Prior Informed Consent Form, in compliance with the ethical precepts of ethnobiological research of the International Society for Ethnobiology Code of Ethics [41]. The research was registered for associated traditional knowledge access in the National System for the Management of Genetic Heritage and Associated Traditional Knowledge (SISGEN) is under number A315C86. The authorization for collection of botanical material is registered by the number 6120635 in the Brazilian Biodiversity Authorization and Information System (SISBIO).

Data were collected through open and semi-structured interviews [42, 43] with key participants from each indigenous land, botanical collection, and identification of the plants mentioned in the interviews. We used a non-probabilistic sampling for the interviews, including adults (over 18 years) who were the most involved with medicinal or healing use of plants, teachers, political or spiritual leaders, and those who usually participate in plant exchanges. We also respected social status within the villages, and we interviewed a maximum of five people in each village, also considering the optimization of time and resources available for the study [44]. The semi-structured interviews had two parts: the first referred to the cultural and social characteristics that encourage or do not encourage the exchange of plants and to the person's perception of the environment, including the use of plants in traditional ceremonies, if she/he often visits other villages, if he/she was kinship related to people from the visited villages, and how important the forest environments are (full interview protocols are available upon request). The second part of the interview addressed the plants used for medicinal or healing purposes, whether they are native or exotic in the village, as well as which plants were exchanged and with which villages these exchanges occurred.

Plant collection was conducted according to Ming [45], and we had the support of botanical experts for the identification of fertile and vegetative material. Fertile vouchers were deposited in the UFSC FLOR Herbarium under numbers 66299 to 66319 and 67486 to 67487. For plants whose collection was not possible, we made photographic records to check the possible botanical identification.

### Data organization and analysis

Data were analyzed qualitatively and by calculating percentages of responses. Parts of the responses of the interviewees were transcribed, and those speeches inserted in the results were identified by the interviewee number, followed by the name of the *Tekoá* and Indigenous Land. Due to the different number of interviewees per village, the analysis of plant exchanges considered each village as a sampling unit. The exchanges made between the villages were analyzed through a bipartite network, in which we relate the visited villages with the villages that were mentioned in the plant exchanges. We used the package bipartite of the R platform Rx64 3.4.1 [46]. We analyzed the origins and destinations of the interchanged plants to observe the movements between phytophysiognomies and the Guarani contributions to the ecological configurations of the vegetation and the selection of species.

Reported medicinal plant applications were separated into use categories according to the worldwide standard for disease classification of World Health Organization (WHO) [47]. The idea of using this classification is not to standardize the traditional uses of plants, which go far beyond the WHO [45] classification, but to show the medicinal contexts in which plants are being applied and exchanged [48]. We also considered that due to the context of use, some plants do not have a discrete distinction between medicinal and healing, as we defined in this study.

## Results

Twelve key interviewees participated in the research: four women and eight men (five people at Cantagalo, two at Morro dos Cavalos, and one person in each of the other Indigenous Lands). Five interviewees needed the help of a Portuguese-Guarani translator. The other interviews were conducted in Portuguese, when interviewees were both Guarani and Portuguese speakers. Their ages varied from 30 to 69 years. Often, during the conversations, other people were present, sharing their knowledge about plant uses. Respecting the social context, we did not exclude them at these moments.

These key interviewees reported the use of 49 plant species of 27 botanical families (Table 2), and more than 86% of these species are native. The most representative family was Asteraceae with 6 species, followed by Myrtaceae and Fabaceae with 5 species each. The highest number of citations was for *Jacaranda micrantha* and *Tabernaemontana catharinensis*, each mentioned in three different villages. Four species were cited in two different villages: *Schinus terebinthifolius*, *Luehea divaricata*, *Cabralea canjerana*, and *Petiveria alliacea*. The other species were mentioned in only one village. Thus, there were 58 total citations of the 49 plants.

**Table 2:** List of species reported in 12 interviews in Mbya Guarani villages in southern Brazil

Family/Species	*Guarani name*	Portuguese name	Quotes/village
**Amaranthaceae**			
*Chenopodium* sp.*	*Ka’a ré*	–	1
**Anacardiaceae**			
*Schinus terebinthifolius* Raddi*	*Yrywajá orembiu*	Aroeira mansa; pimenta rosa	2
**Apocynaceae**			
*Tabernaemontana catharinensis* A. DC.*	*Pipi guaxu*	Jasmim-cata-vento; forquilinha	3
**Aquifoliaceae**			
*Ilex paraguariensis* A. St. -Hil.*	*Ka’a*	Erva-mate	1
**Araceae**			
*Philodendron bipinnatifidum* Schott ex Endl.*	*Ywaimbé*	Guaimbé; cipó-imbé	1
**Asteraceae**			
*Achyrocline satureioides* (Lam.) DC.*	*Ipoty ju va'e*	Macela	1
*Baccharis trinervis* (Lam.) Pers.*	*–*	Japecanga	1
*Calea pinnatifida* (R. Br) Less*	*Yxiporó*	Cipó-flor-de-maria-mole, jasmim-do-mato, quebra-tudo	1
*Lepidaploa balansae* (Chodat) H.Rob.*	*Gajuruguay*	Tatatai	1
*Matricaria chamomilla* L.	*–*	Camomila	1
*Vernonanthura tweedieana* (Baker) H. Rob.*	*–*	Mata-campo	1
**Begoniaceae**			
*Begonia cucullata* Will.*	*Araku ka’a*	Azedinha do brejo	1
**Bignoniaceae**			
*Dolichandra* sp.*	*–*	Unha-de-gato	1
*Jacaranda micrantha* Cham.*	*Para paray*	Caroba	3
**Boraginaceae**			
*Cordia americana* (L.) Gottshling & J.E.Mill.*	*Guajayvi*	Guajuvira	1
*Cordia monosperma* Roem. & Schult.*	*–*	Erva-baleeira	1
*Varronia curassavica* Jacq.*	*–*	Erva-baleeira	1
**Bromeliaceae**			
*Vriesea platynema* Gaudichaud var. platynema*	*–*	–	1
**Cactaceae**			
*Cereus hildmannianus* K.Schum.*	*–*	–	1
**Celastraceae**			
*Maytenus* cf. *ilicifolia* (Schrad.) Planch.*	*Yvyrá poju*	Espinheira santa	1
**Cucurbitaceae**			
*Cayaponia* cf. *palmata* Cogn.*	*–*	Tayuya	1
**Dioscoreaceae**			
*Dioscorea* sp.*	*–*	Salsaparrilha	1
**Euphorbiaceae**			
*Euphorbia pulcherrima* Willd. ex Klotzsch	*–*	Estrela de natal; bico de papagaio	1
*Sapium glandulosum* (L.) Morong*	*Kurupika’y*	Pau-leiteiro	1
**Fabaceae**			
*Cajanus* cf. *cajan* (L.) Millsp.	*–*	Feijão-guandú	1
*Inga virescens* Benth.*	*Inga*	Ingá	1
*Lonchocarpus* cf. *cultratus* (Vell.) Azevedo-Tozzi & H.C.Lima*	*Yvyrá kati*	Rabo-de-bugio	1
*Machaerium stipitatum* (D.C.) Vogel*	*Ixapy'y*	Farinha-seca	1
*Zollernia ilicifolia* (Brongn.) Vogel*	*Yvyra karai*	Falsa espinheira-santa; fura-olho; carapicica-de-folha-lisa	1
**Gesneriaceae**			
*Sinningia douglasii* (Lindl.) Chautems*	*–*	Bata das árvores	1
**Lamiaceae**			
*Plectranthus* cf. *barbatus* Andr.	*Teeraxy poã*	Boldo brasileiro	1
**Lauraceae**			
*Cryptocarya aschersoniana* Mez*	*–*	Canela-fogo	1
**Malvaceae**			
*Luehea divaricata* Mart. & Zucc.*	*Yxonguy*	Açoita cavalo	2
*Sida rhombifolia* L.*	*–*	Guanxuma	1
**Meliaceae**			
*Cabralea canjerana* (Vell.) Mart.*	*Cansarana*	Canjerana	2
*Cedrela fissilis* Vell.*	*Yary*	Cedro rosa	1
**Myrtaceae**			
*Eugenia uniflora* L.*	*Nhanga pity*	Pitangueira	1
*Myrciaria floribunda* (West ex Willd.) O. Berg*	*–*	Cambuíva	1
*Plinia peruviana* (Poir.) Govaerts*	*Ywapuru*	Jaboticabeira	1
*Plinia rivularis* (Cambess.) Rotman*	*Guaporoity*	Guapuriti	1
*Psidium guajava* L.	*Araxa guaxu*	Goiaba	1
**Phytolaccaceae**			
*Petiveria alliacea* L.	*Pipi*	Guiné	2
**Polygalaceae**			
*Polygala paniculata* L.*	*–*	Gelol/Timutu-barba-de-são-Pedro	1
**Rosaceae**			
*Prunus myrtifolia* (L.) Urb.*	*Yvaró*	Pessegueiro do mato	1
*Prunus persica* L.	*–*	Pêssego	1
**Solanaceae**			
*Physalis pubescens* L.*	*Membyraxy poã*	Camapu	1
*Solanum americanum* Mill.*	*Ka’a teí*	Erva-moura; maria pretinha	1
*Solanum mauritianum* Scop.*	*Kavaxinguy*	Cavatinga	1
**Urticaceae**			
*Urera baccifera* (L.) Sand.*	*Pyno*	Urtiga vermelha	1

* = Native species

### Plant exchanges and therapeutic uses

**Fig. 1:**
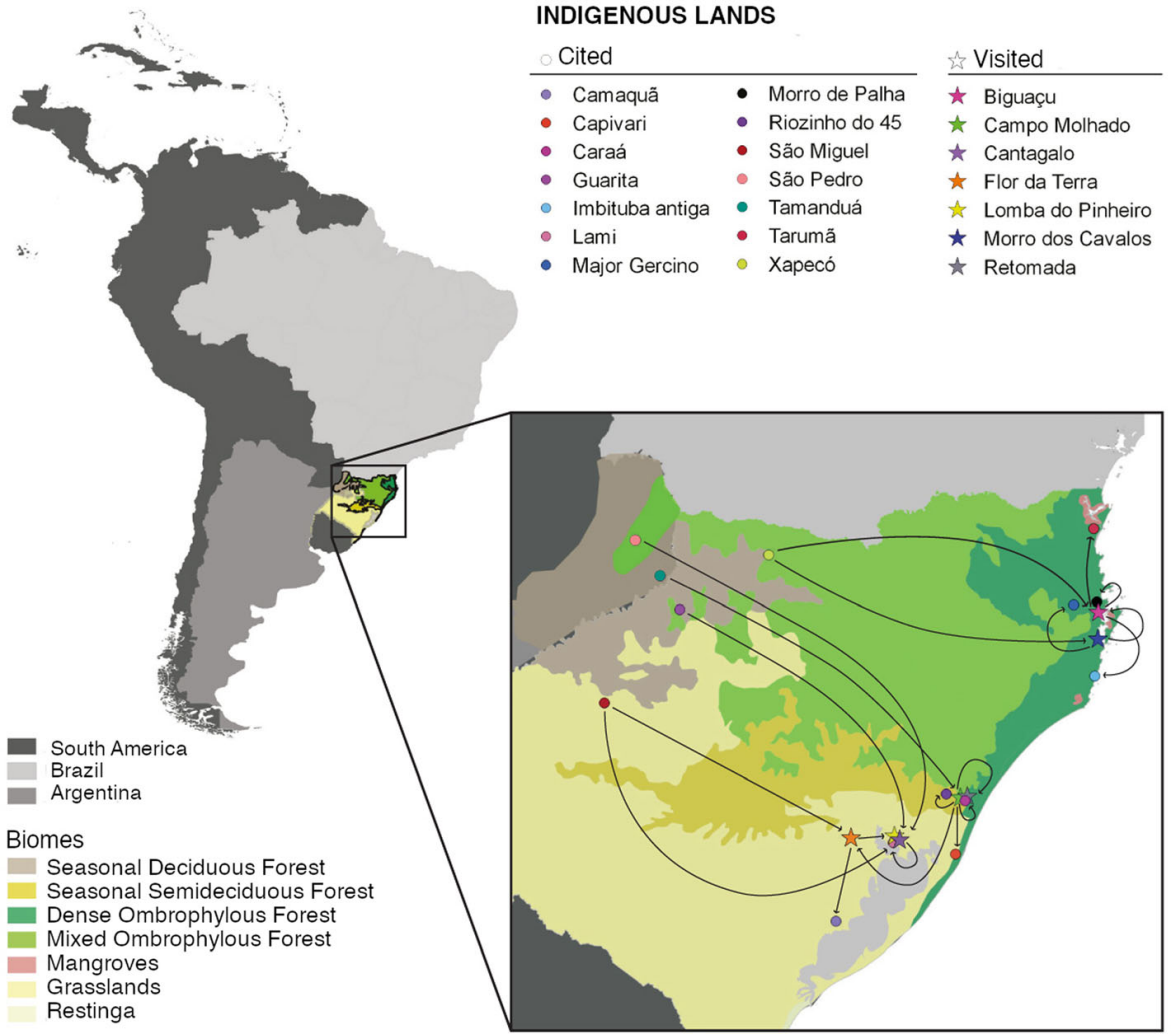
Location of the villages visited in the study (stars) and those with which were exchanges (circles), with the phytophysiognomies in which the villages are present. The arrows indicate the flow of propagules between villages

**Fig. 2:**
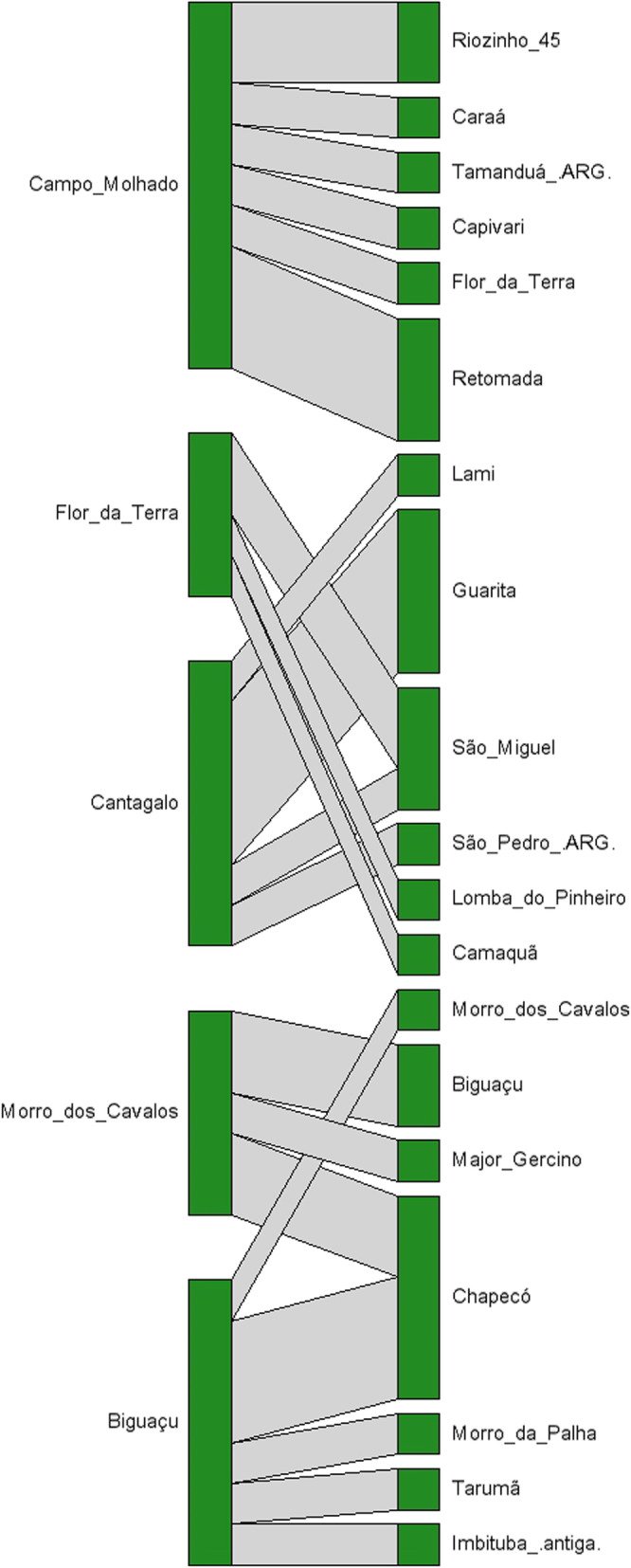
Bipartite network illustrating the exchange of propagules between Guarani villages, built from interviews in seven villages in Santa Catarina and Rio Grande do Sul. In the left column are the villages visited; on the right are the villages with which exchanges took place (ARG indicates that they are villages located in Argentina). The intensity of the connections determines the number of plant changes

The interviewees of the seven villages exchanged plants with 19 villages in the states of Rio Grande do Sul, Santa Catarina, and the province of Misiones in Argentina (Figs. 1 and 2). Of the 58 plant citations (including repeated citations), propagules were exchanged for 46% of these plants (27 plants), summing 33 exchanges. Some plants were exchanged more than once; for example, *Zollernia ilicifolia* was received by one village and was supplied to three other villages, accounting for four exchanges. No exchanges were reported by the interviewees at Retomada and Lomba do Pinheiro, although these villages were indicated in other villages as suppliers or recipients of propagules. For this reason, these two villages were not included in the column of visited villages of the bipartite network (Fig. 2).

The reasons for the visits include the search for seeds and medicines, visiting relatives, political articulations, knowledge exchange, the strengthening of themselves and of the Guarani culture, helping each other, and the well-being associated with travels and visits.

According to interviewee #1 (*Tekoá Ka’aguy Porã*, Retomada), “when visiting relatives, the place is always observed, if it is good for children, if it is healthy”. During these visits, seedlings of medicinal plants and medicine knowledge are also exchanged. Most exchanges take place with plants ready to use, such as the barks or leaves; when the seeds or seedlings are available, they are exchanged, but this does not guarantee that the propagule will be established in the other village because the environments can be very different. Plants are not necessarily exchanged through the mediation of a giver: if the person who visits another village knows which plant is needed, he/she just brings it. Most of the plants received in exchanges are incorporated in the families' homegardens, among other species brought directly from the nearby forests, becoming easily accessible when needed.

Seedlings or vegetative material transported between villages included especially native species, corresponding to 89% of the plants exchanged (24 species). Only three exotic species were exchanged: *Matricaria chamomilla*, *Plectranthus* cf. *barbatus*, and *Cajanus* cf. *cajan*. Among the exchanged plants, *Tabernaemontana catharinensis* was the most cited. Some older exchanges were reported at Morro dos Cavalos, but with the precise origin of the plants unknown, as some *Plinia peruviana* trees brought from Argentina, probably in the 1960s. At least one individual of *Campomanesia* sp*.* and seedlings of *Ilex paraguariensis* were also brought in the past to this village.

Most plants were cultivated (67%) and 33% spontaneous. Trees comprise 52% of the exchanges, herbs 22%, shrubs 15%, and vines 11%. Exchanged medicinal plants are mainly used to treat blood and circulatory system (3); cuts, scars, and skin (6); problems in the joints (1); cancer (1); digestive diseases (5); respiratory (5), genitourinary (3), and feminine cycles (5); contemplating vital functions of the organism, in addition to the healing uses of spiritual importance. Some plants have more than one therapeutic function. For example, according to the chief of Cantagalo, some plants are used to take care of physical and other spiritual diseases. For him, *juruá* (non-indigenous) medicines are used to treat physical issues, but for spiritual illness, only the *Karai* (spiritual leadership) knows how to solve the illnesses through traditional medicine. Guarani establishes complex relationships with the plants depending on the person and the plant, and many physical and spiritual diseases are not split into different categories. For interviewee #4 (*Tekoá Yakã Porã*, Morro dos Cavalos), “The medicinal herb for the Guarani people is sacred, it is not just an herb, a bush, or a leaf, it is a spirit, a brother of ours, who helps in healing, who helps in liberation. So for us the medicinal herbs are very sacred”. For interviewee #3, “Those who work with medicines have to know well which plant to use. There are many remedies, but plants are specific to each thing. There are two types of *yxonguy*, for example, and each has a different application. To be accurate at the time of diagnosis, you need to talk well with the person to know the history of the disease”.

A remarkable topic related to medicinal plant uses is women’s illnesses. According to interviewee #5 (*Tekoá Itaty*, Morro dos Cavalos), there are a number of recommendations during the woman’s gestation period, which should be followed by both parents. These include using plants to maintain the well-being of children and parents. In addition to chamomile, other plants appear in the exchange list whose application is intended for women’s health, such as *Jacaranda micrantha*, *Physalis pubescens*, *Schinus terebinthifolius*, and *Plinia rivularis*. On one of the visits to *Tekoá Nhuu Porã*, we observed the dissatisfaction of one mother who reported that her daughter had to give birth in a hospital, and traditional methods involving herbs could not be performed.

### Mbyá Guarani eco-cultural health and the forest environments

Some species have been described as essential for conducting ceremonies at *Opy* (ceremonial house). The presence of the *Opy* in each *tekoá* is central. The *Opy* is a space for meetings and sharing, for ceremonies, chants, dances, healings, and for the relations with other beings and deities, expressing Guarani cosmology. Within the *Opy*, plants play an essential role, in which plant and forest properties are incorporated. The use of native plants is a priority in ceremonies, including those used to light the sacred fire, such as *Holocalyx balansae* and *Cedrela fissilis. C*. *fissilis* is also used in objects such as the *apyka* for the baptism of children, in baths or infusions, together with *ka'á* (*Ilex paraguariensis*), and with the herbs that will be used and strengthened in prayers. Guarani white corn (*Zea mays*) is central, as well as *ka'á* and *pety* (*Nicotiana tabacum*), to open the concentration, to have spirit, and to maintain the culture. *Pipi* (*Petiveria alliacea*) is used to strengthen the body and mind and in baths for cleaning, as well as *yvaró* (*Prunus brasiliensis*), and to receive spirits well. Many plants used traditionally have magical value, but this information is not widely shared.

The Guarani healing context is intrinsically related to the forest. For interviewee #8 (*Tekoá Jatai*’*ty*, Cantagalo), “We are children of nature, she provides what is necessary to live in it, the elderly always say, it shows the way of how to live". For interviewee #9 (*Tekoá Yvy Poty*, Flor da Terra), “Every indigenous person knows that without nature, they have no conditions, the land strengthens life, and with a lot of forest they have everything: food, health, education, knowledge and guarantees culture”. The perceptions regarding the presence and importance of the woods and forests revealed key ideas such as health, culture, joy, life, food, balance, education, and knowledge.

The presence of forests is directly related to both individual and community good health. Interviewee #3 reinforced the Guarani cosmological view, with the central relationship between health, plants, and forest: “Our body is just like the earth, that's why nature heals us. […] All plants act on the health of the planet itself and maintain the health of all beings. […] A fruit is like medicine; when we eat it, we are healing. This is about health, *Nhanderú* guides our way, whenever we ask from the heart. [...]. Many *juruá* say that the Guarani are losing knowledge of the plants, but the Guarani know that the most valuable plants have been cut. Barks have been used since childhood, and a tree has never died, it still lives 100, 200 years and the person thus has protection”.

The disappearance of the forests and the demarcation of territories were also mentioned, since every indigenous person keeps the ancestral territory in their memory, and for some of them, the idea of limiting a space to live becomes almost incomprehensible. Many *poã* (remedies from the forest) are no longer found within the limited indigenous land, and searching for certain plants that are outside the demarcated territories incurs a risk of being mistaken for a criminal act. According to interviewee #9 (*Tekoá Yyn Moroty Vherá*, Mbiguaçu), “The territory is not restricted to the demarcated area, now, many plants are found outside the area”.

At Campo Molhado indigenous land, which is located in an ecotone, the chief reported the presence of a total of 24 plant species brought to the village that can be currently found only in the forests of Misiones in Argentina. In other villages, the lack or rarity of some important species in traditional medicine include plants such as *ipê* (*Handroanthus* sp.) trees in Lomba do Pinheiro and Mbiguaçu, and *katiguá* (*Trichilia* sp.) and *cipó-guembé* (probably *Philodendron bipinnatifidum*) at Retomada. Up to the time of this study, three of the seven villages had seedling nurseries for reforestation of native species of traditional importance.

## Discussion

### The influence of Guarani plant exchange networks on Atlantic Forest composition

In the Guarani cosmology, the natural world is closely connected with the supernatural world, which often occupies the same spaces [27, 49]. Within this cosmology, the maintenance of botanical knowledge with therapeutic functions is also important [50], as well as the exchanges among villages. The connections between the villages through family and kinship ties facilitate plant exchange networks across the Guarani traditional territory. Our study focused in a limited set of plants, when compared to more extensive studies on Guarani plant knowledge (e.g., [1, 18, 27, 28, 32, 33, 50]) which inventoried hundreds of species, but even with this small set we can perceive the role of Guarani exchanges and mobility contributing to forge the cultural landscape of Atlantic Forest. Our results reveal a small snapshot of the current panorama of the networks that connect the Guarani villages and the exchanges of plants that are still taking place in the Atlantic Forest biome, with emphasis on native species. A caveat of our study is that this snapshot is also limited by our small sample of villages and interviewees in each village, which were guided by Guarani’s availability and by their own indications of who we should interview.

In the exchange network, *Tekoá Nhuu Porã* (Campo Molhado), *Tekoá Yyn Moroty Vherá* (Mbiguaçu), and *Tekoá Jatai’ty* (Cantagalo) stand out with more citations and connections with the other villages mentioned (Figs. 1 and 2). These villages are located closer to the coast, and the plant exchanges revealed links with villages that are in the west part of Rio Grande do Sul and Santa Catarina States, as well as with the region of Misiones, in Argentina, more than 900 km from the Atlantic coast. In those areas, the Alto Uruguai Deciduous Seasonal Forest formations predominate, and the Indigenous Lands keep valuable forest fragments amidst monocultures. In Argentina, there are greater extensions of forest cover. One of these areas is the Yabotí Biosphere Reserve [51], a region identified by the Guarani as a place of reference to find certain floristic elements. Exchanges between these western villages and the Atlantic coast were already observed [27], with the transportation of propagules such as *Chrysophyllum gonocarpum*, *Luehea divaricata*, *Parapiptadaenia rigida*, and *Holocalyx balansae* to the village of Mbiguaçu.

Almost half of the plants exchanged were trees, followed by herbs, similar to what Heineberg and Hanazaki [35] observed among the Laklãnõ-Xokleng (from Jê linguistic group). The plants associated with exchanges have the potential to be incorporated in environments different from those of the place of origin. For example, among the Myrtaceae family—one of the most important in the Atlantic Forest, with several edible fruit species [28]—*Plinia rivularis* was mentioned as an exchanged plant. It was brought from a place with Deciduous Seasonal Forest of upper Uruguay to Flor do Campo village, where the phytophysiognomy is of Semideciduous Forest. The origin of the individual of *Plinia peruviana* mentioned in Cantagalo is unknown since it was cultivated by a former resident. According to Sobral et al. [52], this species is characteristic of the Seasonal Semideciduous Forest and Mixed Ombrophylous Forest, which are forest formations different from the forest at Cantagalo. The dominance of this family in the Atlantic Forest may be related to the management carried out by indigenous peoples in the past, including, for example, enriched areas with Myrtaceae fruit trees in the South region found between the nineteenth and twentieth centuries [28].

Several exchanged plants are related to religious beliefs. Keller et al. [50], citing Cadogan [12], highlighted the complexity of Mbyá Guarani medicine plants related to these beliefs, which include species such as *Tabernaemontana catharinensis.* In the two villages of Rio Grande do Sul state, the seedlings of this species were brought from a village at São Miguel das Missões, *Tekoá Koendju*. This species occurs in the whole Atlantic Forest region [52, 53], but for the interviewees, it is rare, and the individuals present in the studied villages come from the exchanges. In Mbiguaçu, all individuals of *T*. *catharinensis* were planted, brought in the 1980s, but of unknown origin.

Yerba mate, or *ka'a*, *Ilex paraguariensis*, is a species exchanged with cultural and medicinal value for Guarani [54, 55]. A variety of *yerba mate* with a lighter shade on the leaves were brought from Misiones to Campo Molhado indigenous land, and it is different from the one already found there, showing the potential of exchanges to increase the local variability of the species. Individuals of this species are also present in Morro dos Cavalos indigenous land, at Ombrophyllous Dense forest, which is not the attributed distribution for this species (usually occurring above 400 m of altitude [56]). The historical relationship of *yerba mate* with indigenous peoples is not restricted to the Guarani: the species was also consumed among peoples of the Chaco and the Andes, where it does not occur spontaneously [55]. The name *mate* is even derived from the Quechua word *mati*, which means the gourd or *porongo* where the drink is traditionally consumed. This leads us to an interethnic network of relationships in which people probably exchanged and cultivated the plant [55]. Oliveira and Esselin [55], based on Posey [57], considered the plant as semidomesticated because it is intentionally managed. If this management had not already taken place by the natives, it would have been more difficult to expand the herbs for economic exploitation, as occurred in the nineteenth century [55].

Another highly esteemed species is *Cedrela fissilis*, *yary*, which is considered a medicine of sacred value [1, 16, 50]. It is a well-distributed species in South America and is a threatened tree species, with a vulnerable status [53] due to logging and suppression of the Atlantic Forest. Given its cultural importance, it is likely that the Guarani historically contributed to the dispersion of this species through their exchanges, contributing to its distribution in the Atlantic Forest landscape.

*Petiveria alliacea*, although a naturalized species, is another example of the Guarani influence on species composition in the southern Atlantic Forest. At Cantagalo, it was brought from the nearby village of Lami. Galante [1], Keller et al. [50], and Bueno et al. [58] mentioned the traditional use of the species by the Mbyá-Guarani of Misiones (Argentina), the Kaiowá and Guarani of Mato Grosso do Sul, and the Guarani of São Paulo, respectively. This plant is also used by other indigenous groups, such as the Ka’apor of Maranhão, also from the Tupi group [59].

*Solanum mauritianum* and *Urera baccifera* have a wide distribution both in the Atlantic Forest and in other Brazilian biomes, respectively [53]. Both species were brought from the deciduous to semideciduous forest at Cantagalo and are mentioned in other studies with the Guarani [1, 16, 27], showing once again that the Guarani exchanges may have contributed historically to the composition of different phytophysiognomies.

Some species are naturally occurring in the phytophysiognomies of indigenous lands; however, they can be locally rare or absent. For example, *Philodendron bipinnatifidum* is one of the plants that are named at *Opy*, so that their *ja* (owner) authorizes their use [1]. It is native to all forest formations in southeastern Brazil [56]. However, in Morro dos Cavalos, there was only one known individual, a fact that also motivated the exchanges. *Luehea divaricata* is another important species in Guarani medicine [27]. The species is present in the cerrado (Brazilian savannah) and in all formations of the Atlantic Forest [52]. Despite this, in Campo Molhado, it was reported as not available spontaneously, which motivated the exchange. Oliveira [27] reported the absence of the plant in Mbiguaçu and the request of people from this village to purchase seedlings for planting.

Guarani exchanges can also contribute to the distribution of non-native plants. Three species exchanged were probably introduced in Brazil in the colonial period [56]. These plants were also recorded in studies by Oliveira [27] for *Plectranthus barbatus* among the Guarani of Mbiguaçu, Noelli [18] for *Matricaria chamomilla*, and Cossio [16] for *Cajanus cajan*.

Additionally, the exchanges of parts of plants with no propagative potential, such as for *Inga virescens* taken from Campo Molhado to a village in the Pampa biome, reflect the distribution of knowledge and the broad spectrum that the relationship Guarani plants reach. This species is related to altitude forests, being better distributed in the Mixed Rain Forest [60] and absent in Pampa.

Oliveira [27] discussed possible migratory routes that contributed to the transport of species of flora from seasonal forests from the interior of the South American continent to the Atlantic coast. Klein [61] elaborated a list with species that would be characteristic of these routes, some of them mentioned in our study: *para paray*—*Jacaranda micrantha*, *yxonguy*—*Luehea divaricata*, and *pipi*—*Petiveria alliacea* [27]. Factors such as climatic fluctuations could have favored seasonal forests so that certain species could reach the Atlantic coast [62], but Oliveira [27] states that the routes proposed by Klein [61] overlap with the archeological sites of Guarani presence in southern Brazil, where many villages are also present today [27].

### Guarani family, extended family

We found that more than half of the plant exchanges occurred between villages whose interviewees had close relatives, but there were also exchanges between villages with no close kinship ties. The concept of extended family is “the most widespread sociological model” in Amerindian social organizations [22, 63]. For Guarani, this organization is composed of several nuclear families (women and men who live together and their children) united by kinship and affinity relationships. Thus, an extended Guarani family can be made up of the wife (or a group of sisters) and her husband, the daughters married to their sons-in-law, unmarried children, and their daughters’ children. In these bonds, the “blood” relatives are called *retarã*, affinity relatives are the *towadjá*, and the aggregates also recognized as relatives by the bonds with the host family are the *guapepó*. The extended family can include many domestic groups spread over several villages [20, 22].

Being on the move is a way for the individual to maintain health and happiness [25]. In this sphere, the sense of being in the Guarani world is added, such as the search for *Yvy Marã’ey*, the Land without Evils, and the *mborayu*, or reciprocity [20, 49]. These elements favor the exchange of plants between people and villages, which end up circulating in different phytophysiognomies while strengthening social bonds, kinship, and affinity relationships [22].

### Teko porã, well-being, and eco-cultural health within Atlantic Forest cultural landscape

Within their extended territory, all *tekoá* are connected and, through their geographical distribution, represent the support and structure of the Guarani world [64]. Religiosity permeates the Guarani daily life, and *Opy* holds the position of the social, political, religious, and educational center of the village [21, 22, 25].

For the well-being and maintenance of Guarani customary practices, forest environments are essential, which permeate the reasons for migration in search of a good place to settle. Forest environments provide the essentials for health and happiness, good water and land, a source of food and medicines, and direct contact with deities. Taking care of rivers, land, and forests is part of the individual and collective health that involves not only humans but also those who share these spaces in the natural and supernatural worlds, and these environments must be maintained for those that will come later because so did the ancestors [49, 65].

Thus, Guarani migrations and exchanges of plants are intertwined with the historical ecology of Atlantic Forest not only for material and utilitarian purposes of plant species, but due to their whole notion of eco-cultural health and well-being. Although historically the Guarani have occupied the lower lands of the Atlantic Forest [66], in the archeological record, there are Guarani sites in the three southern states of Brazil in the area of mixed rain forest and forest transition areas [10]. Villages present in transition areas of phytophysiognomies (as shown in Fig. 1) can contribute to the Guarani interaction with a diversity of species and emphasize the effects of indigenous management of the Atlantic Forest landscape (see also Pereira Cruz et al. [30]). For Guarani people, dreams and visions of older people often guide decisions when considering places to live, relating the natural environments to the cosmology of the Guarani way of life [27, 65].

There are essential plants used in rituals that the Guarani seek to maintain. The small spaces in which indigenous lands are located, especially in southern Brazil, impose challenges to access and collect plants, such as orchids at indigenous lands near Porto Alegre and at Morro dos Cavalos [67], and medicinal plants [68]. The interest in the presence of plants intended for women’s health demonstrates, for example, the concern of communities to take care of everything that involves women’s cycles and is also a way of maintaining traditional practices amidst social and environmental changes, without relying on medical assistance from *juruá kuery* [69, 70]. Noelli [18] emphasizes in this sense the flexibility and structure of the Guarani medical system, which over the past centuries has sought the efficacy of both native and introduced plants to combat the entire arsenal of previously unknown diseases to which they were subjected, such as influenza, smallpox, measles, malaria, typhus, yellow fever, venereal diseases, and tuberculosis.

Historically, the Guarani have demonstrated their flexibility in dealing with the natural resources they have, in parallel with their sociocultural unity. The search for the necessary conditions for well-being and the understanding of eco-cultural health with an intrinsic link between health and forest also support the process of domestication of the landscape [31, 71, 72]. Historical and continuous interactions with forests maintain important elements in the environment for cultural continuity in a healthy and safe way. Plant exchanges and the management of certain species can also provide information about possible domestication processes that may occur at different levels [27, 28, 30] and contribute to the continuous genesis of cultural landscapes of Atlantic forests.

## Conclusions

Almost half of the plants reported were involved in some exchange between villages, which marks an intense movement of plants in the Atlantic Forest to strengthen health and culture and enrich the forest environments. In this sense, the Guarani are enriching their territories with species related to their traditional pharmacopoeia, both for physical and spiritual health. This process is related to the traditional mobility inherent to the Guarani people, also with contributions of the partnerships that have been established between the villages and educational institutions, the civil community, and the creation and maintenance of nurseries in the villages. We observed through this flow of plants and knowledge the real possibility of species to be incorporated in phytophysiognomies different from those in which they are usually described, such as, for example, the species characteristic of the Seasonal Semideciduous, and Mixed Ombrophyllous forests taken to Ombrophyllous Dense forest. For Pereira et al. [28], the Guarani contribution to the ecological configurations of these environments, such as favoring some species, is still an open topic for study, which brings us to millennia of human influence in the Neotropics. Currently, indigeneity in landscapes [71] is being registered and mapped, as territorial management plans have been developed in indigenous lands, adapting traditional botanical and ecological knowledge to contemporary reality.

The presence of indigenous communities, through the conscious management of the territory, helps to maintain species of ecological and cultural importance [29], sometimes threatened, being extremely important for the conservation of the biodiversity of the Atlantic Forest. This also opens the debate about what is native and exotic in the Guarani conception, especially when considering the scope of the original Guarani territory and their historical interaction with the Atlantic Forest landscape. Many species have been managed and transported in the past, and as we have seen, it is a process that is constantly ongoing. The plants that are migrating among very different phytophysiognomies, associated with human action, are related to the historical management and selection of important species that characterize landscape transformations, in addition to the natural adaptation processes that plants develop.

Recognizing and valuing Guarani knowledge and practices can help restore and conserve natural environments, as well as collaborate for health in a broader way of understanding, as well as develop more sustainable management practices, in addition to providing a better understanding of occupation of the Atlantic Forest in its southern portion.

The Atlantic Forest is a sacred and ancestral territory of immense cultural and environmental value to the world. It is essential to implement public policies that contemplate the safeguarding of these spaces, with the real participation of the people who live in and manage it for longer timespans, such as the Guarani people.

## Data Availability

The data used to support the findings of this study are available from the corresponding author upon reasonable request.
